# Forensic Validation of the 95K SNP Panel and the Parabon Fx Forensic Analysis Platform for Identification of US Military Unknowns Using Extended Kinship Inference

**DOI:** 10.3390/genes17030306

**Published:** 2026-03-03

**Authors:** Jacqueline Tyler Thomas, Courtney L. Cavagnino, Kimberly Sturk-Andreaggi, Ellen M. Greytak, Julie A. Demarest, Suzanne M. Barritt-Ross, Timothy P. McMahon, Charla Marshall

**Affiliations:** 1 Armed Forces Medical Examiner System’s Armed Forces DNA Identification Laboratory (AFMES-AFDIL), Dover Air Force Base, Dover, DE 19902, USA; courtney.l.cavagnino.ctr@health.mil (C.L.C.); julie.a.demarest.ctr@health.mil (J.A.D.); suzanne.m.barritt-ross.civ@health.mil (S.M.B.-R.); timothy.p.mcmahon10.civ@health.mil (T.P.M.); , formerly; 2 SNA International LLC, Contractor Supporting the Armed Forces Medical Examiner System, Alexandria, VA 22314, USA; 3 Parabon NanoLabs, Inc., Reston, VA 20190, USA; ellen@parabon.com; 4 Forensic Science Program, The Pennsylvania State University, State College, PA 16801, USA

**Keywords:** single nucleotide polymorphism (SNP), kinship analysis, next generation sequencing (NGS), massively parallel sequencing (MPS), bone, skeletal remains, forensic DNA, human identification, degraded DNA

## Abstract

Background/Objectives: To identify US military unknowns, the Armed Forces Medical Examiner System’s Armed Forces DNA Identification Laboratory has historically relied upon mitochondrial DNA and Y-chromosomal short tandem repeat testing. Where no appropriate family reference sample (FRS) is available or skeletal samples are degraded, autosomal single nucleotide polymorphism (SNP) testing with next-generation sequencing could assist. Methods: A method utilizing hybridization capture enrichment of a 95,000 (95K) SNP panel, amenable to FRS and extremely challenging samples, was validated. The Parabon Fx Forensic Analysis Platform was used for analysis and extended kinship inference. Skeletal samples (*n* = 65) and associated FRS (*n* = 64) were selected for a performance evaluation and case-type sample study. Results: Considering FRS with ≥7 ng DNA input into library preparation, 94% yielded ≥66,320 SNPs at ≥5X coverage. SNP recovery for skeletal samples at ≥1X coverage ranged from 5 to 94,197 SNPs, averaging 40,770 SNPs. When skeletal samples resulted in ≥13,000 SNPs, the most likely relationship category was consistent with the expected relationship. A log10 likelihood ratio of ≥4 and a posterior probability of ≥99.99% were established as thresholds for strong statistical support, and 87% of inferences met these thresholds while 13% were considered inconclusive. Pairwise kinship inference between unrelated individuals yielded an unrelated result in 85% of comparisons, 66% with strong statistical support. There were 170 instances of false positive 4th degree relationship inferences with strong statistical support. All false positives involved skeletal samples from individuals of admixed ancestry. Conclusions: With this approach, autosomal SNP testing can result in reliable kinship inferences between related individuals out to 3rd, and in some cases 4th, degree relationships, increasing the scope of eligible FRS to aid in identifications.

## 1. Introduction

The mission of the Armed Forces Medical Examiner System’s Armed Forces DNA Identification Laboratory (AFMES-AFDIL) is to identify fallen and missing United States service members (SMs). The Past Accounting Section of the AFMES-AFDIL supports the Defense POW/MIA Accounting Agency (DPAA) in its efforts to identify unknowns from military cemeteries, unilateral turnovers, and field excavations. DNA samples from family members are used as references to accomplish this goal. Postmortem treatment of human remains during World War II (WWII) and the Korean War often involved preservative chemicals, including formaldehyde and fungicides, which resulted in a high degree of fragmentation and DNA damage [1,2]. Historically, the AFMES-AFDIL has used traditional forensic DNA methods, such as autosomal and Y-chromosomal short tandem repeat (STR) typing, as well as mitochondrial DNA (mtDNA) control region sequencing, to assist in identifications. Despite advances in extraction methods and improvements in the sensitivity of commercial STR kits over time, these standard forensic approaches are not sufficient for human identification in many DPAA cases due to severe DNA degradation and other complicating factors, such as environmental DNA contamination. A mitochondrial genome (mitogenome) hybridization capture enrichment method with next-generation sequencing (NGS), also known as massively parallel sequencing, was validated in 2016 according to forensic quality assurance standards [3,4]. This method increased the success rate of the AFMES-AFDIL’s most challenging skeletal samples from ~6% to ~60% [1,4,5]. It also created a laboratory processing framework that could be easily adapted for additional DNA targets, such as large nuclear single nucleotide polymorphism (SNP) panels. Such processing of thousands to millions of SNPs would allow for the use of family members beyond 1st degree relatives, such as paternal nieces, who are not eligible for relationship testing with traditional forensic methods [6]. Additionally, in scenarios where common mitogenome haplotypes may represent multiple SMs, autosomal SNPs can be used for individualization, reassociation of remains, and/or comparison to family reference samples (FRS) to support an identification.

Given the need for novel SNP tools that would be amenable to DPAA cases, a Department of Defense research and development contract was awarded to Parabon NanoLabs (Reston, VA, USA) in 2015 to develop a nuclear SNP panel and an analysis software platform for extended kinship inference. After several rounds of collaborative testing between the AFMES-AFDIL and Parabon NanoLabs, a final method that adapted ancient DNA analyses for relationship inference was shown to be suitable for DPAA samples [7]. This method relied on custom SNP capture panels with tens of thousands of markers analyzed with a genotype likelihood (GL) approach and calculation of kinship coefficients for kinship inferences from very low coverage (1X) sequence data based on NgsRelate [8]. The GL approach utilizes SNP coverage data, base quality scores, and DNA damage (i.e., cytosine deamination) pattern information to calculate the likelihood of each possible genotype, rather than attempting to call genotypes, as SNPs may only be covered by a single read. The GLs are combined with reference population allele frequencies and expected allele sharing distributions to infer the most likely relationship between two samples. While NgsRelate uses Expectation Maximization to find the identical by descent (IBD) proportions (i.e., IBD0, IBD1, IBD2) that maximize the likelihood of the data, Parabon Fx explicitly calculates likelihoods under a specific set of IBD proportions that correspond to particular relationship hypotheses (e.g., 0.25, 0.5, and 0.25, respectively, for full siblings). Parabon Fx also employs hypothesis testing, generating likelihood ratios (versus unrelated) and posterior probabilities for all tested relationships. Though several SNP panels were tested over the course of the research and development process, the proof-of-concept study described in Gorden et al. focused on the final two SNP panels, which comprised 25,000 SNPs (25K) and 95,000 SNPs (95K). Both panels were subsets of the SNPs included in the Infinium CytoSNP-850K v1.1 BeadChip (Illumina, San Diego, CA, USA) SNPs [7]. Panels of this size were intended for searching the internal AFMES-AFDIL FRS database of close relatives of missing SMs (primarily 1st, 2nd, and 3rd degree), rather than a one-to-many search against public DNA databases as with Forensic Investigative Genetic Genealogy (FIGG). The 25K and 95K SNP data analysis was completed in the Parabon Fx Forensic Analysis Platform (Parabon Fx), which allows for capture-to-array and capture-to-capture comparisons. The proof-of-concept study involved the evaluation of the two panels using a set of non-probative DPAA case samples from previously identified SMs and their associated FRS. A comparable proportion of SNPs was recovered with both panels, resulting in more SNPs using the 95K panel for case samples [7]. Though Parabon Fx initially assumed marker independence like NgsRelate [8], analysis of the proof-of-concept data revealed that accounting for linkage disequilibrium (LD) was necessary for accurate kinship inferences. This was accomplished via dynamic pruning of the SNP with lower coverage from each pair of SNPs in LD. Genetic linkage was not accounted for. However, Tillmar and Kling found that analyzing simulated 95K SNP data with an approach analogous to Parabon Fx (NgsRelate, accounting for LD but not linkage) performed comparably to the likelihood ratio (LR) approach that accounted for linkage [9]. Based on the proof-of-concept study [7], the Parabon Fx software was found to successfully infer kinship out to 4th degree (e.g., first cousin once removed) with low coverage SNP data using the GL approach and dynamic LD pruning. This finding is supported by the Tillmar and Kling study [9], which demonstrated that the Parabon Fx-type approach (i.e., NgsRelate with LD pruning of the 95K panel) is unlikely to result in relatives appearing more closely related than simulated for 2nd cousin (5th degree) and closer relationships. In addition, the 95K panel has been employed by the AFMES-AFDIL in numerous historical cases, including one that involved Washington family skeletal samples from the Harewood Cemetery in Charles Town, West Virginia [10], further supporting its effectiveness with low-quality samples.

The 95K panel, due to its improved performance over the 25K panel in the Gorden et al. study [7], was selected for validation for AFMES-AFDIL casework use. The SNPs included in the 95K panel have been previously characterized in the 1000 Genomes Project [11] and the International HapMap Project [12]; therefore, marker characterization was not required, even though this specific panel of SNPs is novel to forensics. The underlying scientific principles were previously published in the aforementioned studies [7,10]. Prior to initiating the developmental validation, several optimization studies were completed to fine-tune the laboratory protocol. These included a study to minimize EDTA inhibition of enzymatic fragmentation using a component of the KAPA HyperPlus Kit (Roche Sequencing, Pleasanton, CA, USA), and an assessment of the number of post-capture PCR cycles needed for FRS libraries. The validation was then initiated in 2023 and completed in 2024 according to the 2020 version of the Federal Bureau of Investigation Quality Assurance Standards. The following studies were included in the validation using both low-quality skeletal samples and high-quality reference-type samples: precision and accuracy (repeatability and reproducibility), sensitivity, NGS multiplexing, species specificity, contamination, stability, population studies, mixtures, and case-type samples, as well as the requirements for validating new software (including functional and reliability testing). A subset of the validation studies is discussed here. The sensitivity and contamination studies are summarized as a performance evaluation. However, the primary focus is the case-type sample study with kinship inference between previously identified SMs and known FRS.

## 2. Materials and Methods

The following lab processing and analyses were completed in-house at the AFMES-AFDIL.

### 2.1. Sample Selection

Both skeletal samples and FRS were necessary to complete the studies discussed. A total of 31 previously identified SMs were represented by 52 skeletal elements and 61 libraries. Some libraries were sequenced at more than one multiplexing level, resulting in 65 sequence data files for analysis. The skeletal samples spanned case contexts and varied in DNA quality (based on prior mtDNA testing), representative of typical AFMES-AFDIL casework [1,2,5]. All samples were approximately 80 years postmortem. A total of 55 individuals were included as reference-quality samples, represented by 64 libraries (each sequenced once), resulting in 64 sequence data files. All 55 individuals were family references, associated with 27 of the 31 SMs. The data selected for each study were based on the needs for that study, as indicated below. Tables detailing sequence data files included in each study can be found in the Supplementary Materials (Tables S1 and S2).

#### 2.1.1. Performance Evaluation

The performance of both skeletal samples and FRS was evaluated with sensitivity and contamination studies. Both sample types ranged in quality and were representative of casework. A total of 54 sequence data files (from 47 skeletal samples and 28 SMs) were selected for the sensitivity study. These were chosen to represent each library only once and included sequence data files with 8 million to 40 million total sequencing reads from 95K-captured libraries. This range of total reads was selected based on typical NGS multiplexing levels for casework processing of skeletal samples. All 64 FRS libraries/sequence data files were included in the reference sample assessment. Control blanks, i.e., reagent blanks (RBs) and negative controls (NCs), processed throughout the validation were analyzed for a contamination assessment. Results from these samples and controls were utilized to set the performance criteria applied during sample selection for the case-type sample study.

#### 2.1.2. Case-Type Sample Study

Sequence data files from various skeletal elements, representing multiple SMs, were selected for the case-type sample study based on performance criteria, including a minimum SNP recovery threshold and confirmation as single source. Some sequence data files were included in the performance evaluation, while others were not. The skeletal samples varied in both DNA quality and biogeographical ancestry, representative of typical AFMES-AFDIL casework [1,2,5]. All samples had associated FRS available for SNP testing.

FRS sequence data files were also selected for the case-type sample study based on performance criteria, including a minimum SNP recovery threshold, and were confirmed to be single source. All FRS selected were also included in the performance evaluation. Each FRS represented a different individual. FRS tested at the AFMES-AFDIL are known to vary in DNA quantity and quality due to time since collection (up to 30 years). Additionally, they are often self-collected rather than being collected with the assistance of a trained individual. This variability was represented in the FRS selected for testing. FRS were saliva samples on sterile cotton swabs (Puritan Medical Products, Guilford, ME, USA) or Bode Buccal DNA Collectors (Bode Technology, Lorton, VA, USA). All FRS donors provided informed consent for their DNA sample to be used for research and quality improvement protocols (Defense Health Agency Office of Research Protections, Protocol # DHQ-20-2073, 20 November 2020).

A range of relationships between the SM and FRS was represented. Parabon Fx can infer relationships out to 4th degree using the GL approach, which is the approach employed for the kinship capture-to-capture function. While the majority of relationships fell in the 1st to 4th degree range, more distant relationships were included to assess the software’s inference in these scenarios.

### 2.2. DNA Extraction and Repair

#### 2.2.1. FRS

FRS extraction was carried out in a high-copy pre-PCR laboratory. DNA extraction was completed on the EZ1 Advanced XL (QIAGEN, Hilden, Germany) using the EZ1 DNA Investigator Kit (QIAGEN) according to manufacturer recommendations, with elution in 50 µL TE (included in the EZ1 reagent cartridge). If no starting substrate was available for DNA extraction, a 40 to 50 µL aliquot of a previously generated DNA extract was used. Previously generated DNA extracts were created via an automated DNA IQ extraction (Promega Corporation, Madison, WI, USA), with a 150 µL elution in 10 mM Tris HCl, pH 8.5 buffer (Tris HCl). No DNA repair was performed for FRS extracts.

#### 2.2.2. Skeletal Samples

Preparation, DNA extraction, and purification of skeletal samples were carried out in a pre-PCR laboratory dedicated to low template and degraded DNA testing. The outer surfaces of bones were cleaned with a Dremel drill (Bosch, Stuttgart, Germany), washed in water and ethanol, and allowed to dry. Bone fragments ranging from 0.2 to 0.5 g were then powdered with a Waring blender cup (Waring, Torrington, CT, USA) [5]. Samples underwent a non-organic DNA extraction and purification method [13], which is based on the Dabney method [14,15]. The bone powder was demineralized overnight in 4 mL of Dabney extraction buffer (0.46 M EDTA and 0.051% Tween 20, pH 8.0) with 200 µL of proteinase K (20 mg/mL) in a 56 °C incubator-shaker. DNA was then purified using High Pure Viral Spin Column Extender Assemblies (Roche Sequencing). To accomplish this, the 4 mL lysate was combined with 40 mL of Buffer PB (QIAGEN) to maintain a 10X ratio of Buffer PB to lysate, which improves the recovery of ultrashort DNA fragments. DNA was bound to the silica spin column, washed twice with Buffer PE (QIAGEN), and eluted with 50 µL sterile 10 mM Tris, 0.1 mM EDTA, pH 7.5 buffer (Tris-EDTA). An RB was initiated with each extraction set and processed along with the samples. Purified DNA extracts underwent DNA repair with the USER enzyme (NEB, Ipswich, MA, USA) for one hour at 37 °C, followed by MinElute (QIAGEN) purification using a 10X Buffer PB-to-DNA extract volume ratio and elution with 55 µL of Tris-EDTA.

### 2.3. Quantification and Library Preparation

DNA from FRS extracts and repaired skeletal sample extracts was quantified with the Qubit 2.0 or Qubit 3.0 (Thermo Fisher Scientific, Waltham, MA, USA) using the dsDNA BR or dsDNA HS Kit (Thermo Fisher Scientific).

#### 2.3.1. FRS

FRS libraries were prepared with the KAPA HyperPlus Kit, which includes an enzymatic fragmentation step. A positive control (PC) and an NC were initiated at library preparation with each sample set, which included 4 to 11 FRS and a single RB. The PC was 45 ng of 2800 M cell line DNA (Promega Corporation), and the NC was molecular biology-grade water. A 15 min fragmentation at 50 °C was used for all FRS. Due to the EDTA content in the FRS DNA extracts, 5 µL of a 5% Conditioning Solution (supplied with the KAPA HyperPlus Kit) was included in the fragmentation step according to manufacturer recommendations. The addition of the Conditioning Solution reduced the maximum FRS DNA extract input from 35 µL to 30 µL. Indexed adapters were used, either BIOO NextFlex 12 nt single-indexed adapters (PerkinElmer, Shelton, CT, USA; no longer available) or 8 nt IDT unique dual-indexed (UDI) adapters (Integrated DNA Technologies, Coralville, IA, USA). An input of 10 µL of 7.5 µM indexed adapters was used for all reference samples and associated controls. Purification after adapter ligation consisted of a 0.8X AMPure-to-library volume ratio with AMPure XP (Beckman Coulter, Indianapolis, IN, USA) and elution in 20 µL Tris HCl. A 12-cycle library PCR was then completed according to manufacturer recommendations using KAPA HiFi HotStart ReadyMix (Roche Sequencing) and 5 µL of 20 µM (stock) KAPA Illumina Primer Mix (Roche Sequencing). A standard 5X Buffer PB-to-library volume ratio MinElute purification was conducted following library PCR with elution in 25 µL Tris HCl.

#### 2.3.2. Skeletal Samples

While the KAPA HyperPlus Kit was needed for the largely intact DNA of reference samples, skeletal samples tested by the AFMES-AFDIL typically exhibit degradation. Therefore, the KAPA HyperPrep Kit (Roche Sequencing), which does not include a fragmentation step, was used for library preparation of skeletal sample DNA. A maximum input of 50 µL DNA extract was possible with this protocol. A PC and an NC were initiated at library preparation with each sample set, which typically included five skeletal samples and a single RB. The PC was 1 ng of enzymatically fragmented K562 (Promega Corporation), prepared according to [4], and the NC was molecular biology grade water. Library preparation included 8 nt UDI adapters, either KAPA (Roche Sequencing) or IDT (Integrated DNA Technologies). An input of 5 µL of 15 uM indexed adapters was utilized for skeletal samples and associated RBs, unless the input into library preparation was ≤1 ng, where 5 µL of 1.5 µM adapters were used. Adapter concentrations of 1.5 µM were used for associated PCs and NCs. Purification after adapter ligation was a 1.3X AMPure-to-library volume ratio with elution in 20 µL Tris EDTA. A 12-cycle library PCR was then completed according to manufacturer recommendations using KAPA HiFi HotStart Uracil + ReadyMix (Roche Sequencing) and 5 µL of 20 µM (stock) KAPA Illumina Primer Mix (Roche Sequencing). A standard 5X Buffer PB-to-library volume ratio MinElute purification was conducted following library PCR with elution in 25 µL Tris EDTA.

### 2.4. Hybridization Capture Enrichment

Both FRS and skeletal sample libraries were captured using a myBaits kit (Arbor Biosciences, Ann Arbor, MI, USA) and v5 chemistry, per the standard manufacturer protocol. Hybridization capture was completed in a thermal cycler at 62 °C for 16 to 24 h (the range recommended by the manufacturer). FRS libraries were captured with the 95K bait panel only, which contains probes for 94,752 SNPs (Parabon/AFDIL 95K Kinship panel, Design ID D10019SNP95K, Arbor Biosciences) [7]. An aliquot of each skeletal sample library was captured with the same 95K bait panel. However, prior to the 95K capture, a separate aliquot of the skeletal sample library underwent capture with Expert Mito human baits (Human, Modern Global, Arbor Biosciences) as a quality control check. This was used to confirm that the sample library was single source, as identifying a mixture in very low coverage SNP data is not always possible. All captured libraries were split into 15 µL aliquots for two separate 19-cycle PCR reactions using KAPA HiFi HotStart ReadyMix (Roche Sequencing) and 5 µL of 20 µM (stock) KAPA Illumina Primer Mix (Roche Sequencing). Replicate post-capture PCR reactions per sample were combined, and a 5X Buffer PB-to-library volume ratio MinElute purification followed, with elution in 20 µL Tris EDTA.

### 2.5. Captured Library Pooling, Quantification, and Sequencing

Captured libraries were pooled by set in equal volume to create one pool for each processing set. FRS set pools were composed of 4 to 10 FRS, one RB, one NC, and one PC. Skeletal sample set pools were typically composed of seven libraries: five samples, one RB, and one NC. The PCs from the skeletal sample sets were not included in the pools and were omitted from sequencing [4]. Set pools were quantified on the Bioanalyzer 2100 using the 7500 assay (Agilent, Santa Clara, CA, USA). Based on the full base pair range, set pools were diluted to 4 nM. Multiple 4 nM set pools of the same sample type (FRS or skeletal) were combined in equal volume for paired-end sequencing on the NextSeq 550 (Illumina, San Diego, CA, USA). The level of multiplexing varied based on sample type and sequencing kit used (Table 1). Due to the expected fragment size range of FRS libraries (200 to 500 bp), 300-cycle kits (151 + 151 cycles) were used for sequencing to enable longer read lengths. However, the skeletal samples contained degraded DNA with fragments <125 bp [4,13] and would not benefit from the same cycling approach. Therefore, 150-cycle kits (76 + 76 cycles) were utilized to minimize sequencing cost and time. The target loading molarity for all sequencing runs was 1.0 pM with 2.5% PhiX sequencing control (Illumina).

After sequencing, FASTQ files were generated using bcl2fastq2 Conversion Software v2.20 (Illumina) with no lane splitting, resulting in an R1 and an R2 FASTQ file per library.

### 2.6. Data Analysis

Analysis of SNP data was completed using the Parabon Fx Forensic Analysis Platform (version 1.5.1.0300). This software platform was discussed in detail by Gorden et al. [7]. In summary, paired FASTQ files were ingested with the appropriate SNP capture panel. FRS data were aligned to the Genome Reference Consortium human reference genome assembly 38, or GRCh38, and PCR duplicates were removed using the Picard MarkDuplicates tool with the --REMOVE_DUPLICATES parameter set to true [16]. Downstream analyses of FRS alignments utilized only unique paired reads (unpaired reads were ignored), where each read pair was counted as a single read. In order to factor DNA damage into GL calculations for skeletal samples, damage first needed to be assessed. Therefore, to enable this assessment, an alternative alignment was performed in which overlapping pairs from skeletal sample FASTQ files were merged into single reads, and any unmerged reads were discarded. Merged reads were then aligned to the GRCh38, and PCR duplicates were removed using the MarkDuplicates tool [7]. The unique merged reads from this alignment were used to estimate the error rate consistent with cytosine deamination for both the 5’ and 3’ ends [17,18] as well as for downstream analyses of skeletal sample data.

Ancestry was estimated using five global reference populations (African, Native American, East Asian, Central/South Asian, and European) based on Parabon Fx ancestry files. These ancestry files were created from 1000 Genomes Project and International HapMap Project data, as well as other published data for the Native American reference dataset [11,12,19,20,21]. Details of the Parabon Fx ancestry files can be found in Table S3. SNP capture GL profiles were generated with a minimum depth threshold of 1X for both FRS and skeletal samples, which included GL calculations for all possible genotypes. Due to the 1X minimum depth threshold employed, any discussion of SNP recovery is based on a 1X coverage threshold unless otherwise indicated.

Finally, kinship analysis between skeletal samples and FRS was completed utilizing Parabon Fx’s capture-to-capture kinship function. Kinship allele frequency files were selected for each pairwise comparison based on the inferred global ancestry of the skeletal sample. Kinship allele frequency files were developed by Parabon NanoLabs for the five global populations plus two admixed populations, African American and Latino. Kinship allele frequency files for the global populations were created with the same individuals used for the ancestry files. The two admixed kinship allele frequency files were created from separate, admixed individuals in the 1000 Genomes Project and International HapMap Project datasets (Table S3). In this study, FRS and skeletal samples were considered admixed when the highest global ancestry proportion was <90%. If skeletal samples had both African and European ancestry, the African American kinship allele frequency file was utilized, and for those with both Native American and European ancestry, the Latino kinship allele frequency file was utilized.

A user-defined r^2^ threshold of 0.2 (the default value) was utilized for LD pruning for all pairwise comparisons. During kinship analysis, Parabon Fx determines the overlapping SNPs for each skeletal sample and reference sample being compared and prunes the SNP with the lower coverage from each SNP pair in LD. LD is calculated from the full reference dataset (i.e., all individuals utilized to create the ancestry and kinship allele frequency files in Parabon Fx) in Plink v1.9 using the --r2 and --ld-window-r2 commands, with a window of 1000 SNPs. At a threshold of 0.2, any remaining LD should have a negligible effect on the kinship results. Genetic linkage was not accounted for. With the capture-to-capture function, including the GL approach, Parabon Fx calculates likelihoods for the following relationship categories: self, parent–child, full sibling, 2nd degree, 3rd degree, 4th degree, and unrelated. For each relationship category, Parabon Fx reports a log likelihood, an LR versus unrelated, and a posterior probability (PP), as well as the most likely inferred relationship category. The PP is calculated considering all tested relationship categories, including unrelated, and assumes equal priors.

The focus of the case-type sample study was to compare skeletal samples to FRS. Any samples that failed to meet the minimum SNP recovery determined for each sample type in the performance evaluation section were excluded (see criteria established in Section 3.1.1 and Section 3.1.3 for FRS, and Section 3.1.2 and Section 3.1.4 for skeletal samples). Each included skeletal sample was compared to all included FRS. For FRS with multiple extracts, only one sequence data file per FRS was included in the final case-type sample data set for kinship analysis. Likewise, for SMs with multiple bones, libraries, and/or sequencing events, only one sequence data file per bone was included. Each sequence data file represented a unique capture and/or sequencing event, composed of both an R1 and an R2 FASTQ file (due to paired-end sequencing), and no data were combined. In one exception, four different bones from a single SM were included along with two associated FRS who were 6th degree and 8th degree relatives of the SM. Four bones were tested in this scenario to include a range of SNP recoveries and to assess the kinship inference statistics generated for more distant relatives. Other than this exception, skeletal sample-FRS pairs with genetic relatedness ranging from 1st to 4th degree, and varying SNP recoveries, were included in order to test the inference capabilities of the Parabon Fx software. The remaining pairwise comparisons were between unrelated individuals, and thus, a most likely relationship category of unrelated was expected from Parabon Fx. Relationship inferences for which the LR was <10,000 (log10 LR <4) were considered inconclusive based on Gorden et al. [7]. Two PP thresholds were evaluated with the related and unrelated pairs of case-type samples, 95% [7] and 99.99% [22]. Regardless of the LR, if no relationship category met the PP threshold being evaluated, the inference was considered inconclusive.

Mitogenome analysis was completed for all skeletal samples using a custom workflow in the CLC Genomics Workbench v12.0.1 (QIAGEN) with the AFDIL-QIAGEN mtDNA Expert (AQME) plug-in [4,13,23]. Briefly, FASTQ files were imported, and reads were mapped to the revised Cambridge Reference Sequence (rCRS) [24,25] using stringent mapping parameters. A custom de-duplication tool included in the AQME plug-in (that mimics the functionality of MarkDuplicates from SAMTools v1.61.1 [7,26]) was integrated into the workflow to remove PCR duplicates, followed by local realignment to assist in indel alignment. A 5X coverage threshold, with a variant frequency ≥10% and a minimum variant count of three, was required for variant calling. AQME tools were further employed to convert the called variants into a forensic mitogenome profile, including an interpretation range and estimated mtDNA haplogroup [23].

## 3. Results and Discussion

### 3.1. Performance Evaluation

#### 3.1.1. FRS Sensitivity

**Figure 1:**
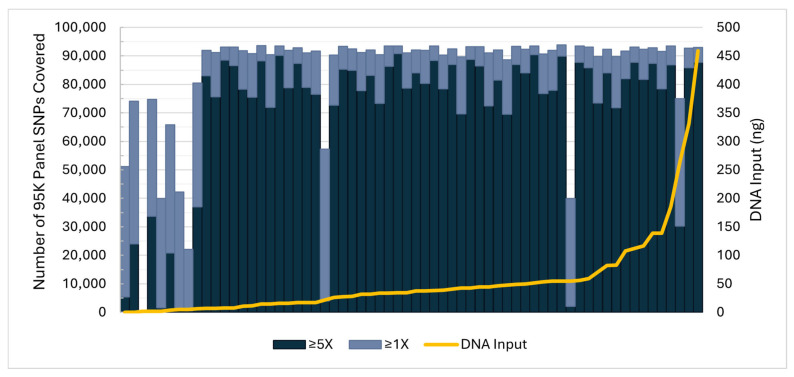
Reference sample total DNA input (right y-axis, gold line) and the number of 95K panel SNPs recovered out of 94,752 (left y-axis) at 1X (light blue) and 5X (dark blue) coverage thresholds in the sensitivity assessment. Each column represents one reference sample sequence data file (*n* = 64).

The 64 reference quality sample libraries, which were prepared with the HyperPlus Kit, resulted in average SNP recoveries of 84,557 (89.2%) at ≥1X, 69,161 (73.0%) at ≥5X, and 58,625 (61.9%) at ≥10X coverage depths. As shown in Figure 1, aside from three exceptions, samples with 1X recoveries below 66,320 (70%) corresponded to FRS with <7 ng total DNA input into library preparation. When considering only FRS with DNA input above 7 ng (*n* = 54), average recoveries increased to 90,159 (95.2%) at ≥1X, 78,131 (82.5%) at ≥5X, and 67,313 (71.0%) at ≥10X coverage depths. It was anticipated that some FRS would result in low DNA quantities due to the time since collection and the collection method (i.e., unsupervised self-collection). The three underperforming samples resulted in fewer than 55% of reads mapped to GRCh38 (compared to an average of 95% mapped for the remaining samples with >7 ng input), suggesting sample quality or processing issues. SNPs covered ≥5X for the library PCs (*n* = 25; 45 ng DNA input) resulted in a minimum of 68,318 (72.1%) and an average of 87,552 (92.4%).

#### 3.1.2. Skeletal Sample Sensitivity Study

**Figure 2:**
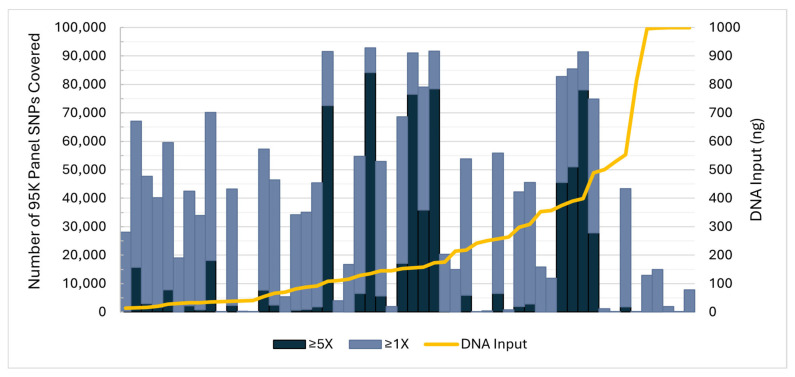
Case sample total DNA input (right y-axis, gold line) and number of 95K panel SNPs recovered out of 94,752 (left y-axis) at 1X (light blue) and 5X (dark blue) coverage thresholds in the sensitivity assessment. Each column represents one skeletal sample sequence data file (*n* = 54).

Unlike FRS, SNP recovery from the 54 skeletal sample sequence data files assessed was not correlated with total DNA input (Figure 2). This was anticipated as many AFMES-AFDIL case samples are known to contain variable proportions of microbial DNA, which contribute to total DNA when using a quantitation method that is not human-specific. Additionally, these samples may contain limited human DNA, which can also be degraded and/or damaged. With such challenging casework samples, additional sequencing output does not necessarily translate to higher coverage of targets (Table S1). Therefore, the number of SNPs recovered is quite variable, difficult to predict, and sample-dependent.

**Figure 3:**
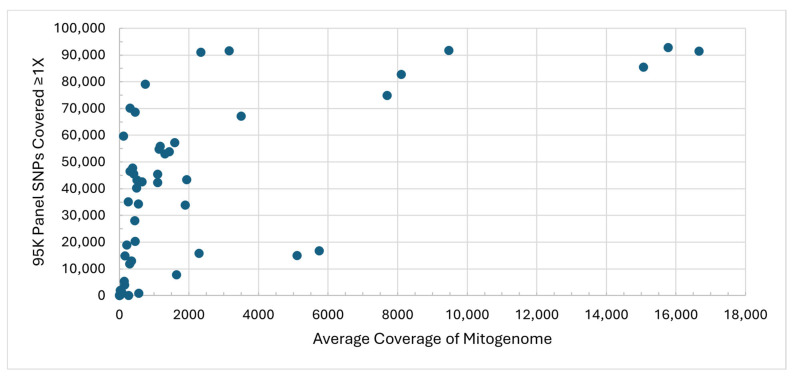
Average coverage of the mitogenome after mitogenome capture versus the number of 95K panel SNPs recovered at a 1X coverage threshold after 95K capture for skeletal sample libraries. Each point represents one skeletal sample sequence data file (*n* = 54).

As skeletal sample libraries were captured twice, with mitogenome baits and separately with the 95K SNP baits, average coverage of the mitogenome was compared with SNP recovery ≥1X for the same 54 sequence data files used for the sensitivity assessment (Figure 3). Unless otherwise indicated, SNP recoveries for skeletal samples will be based on the 1X coverage threshold. Of the 43 sequence data files with >140X average coverage of the mitogenome, only two resulted in fewer than 4000 SNPs, while samples with mitogenome average coverage ≤20X generated a maximum of 231 SNPs. The degree of degradation and microbial DNA content affected relative recoveries, resulting in some samples with lower SNP recoveries than expected based on the mitogenome average coverage. However, the average coverage of the mitogenome does provide insight into which samples and/or libraries are less likely to result in sufficient SNP recoveries for kinship inference and, therefore, would increase efficiency for casework processing.

**Figure 4:**
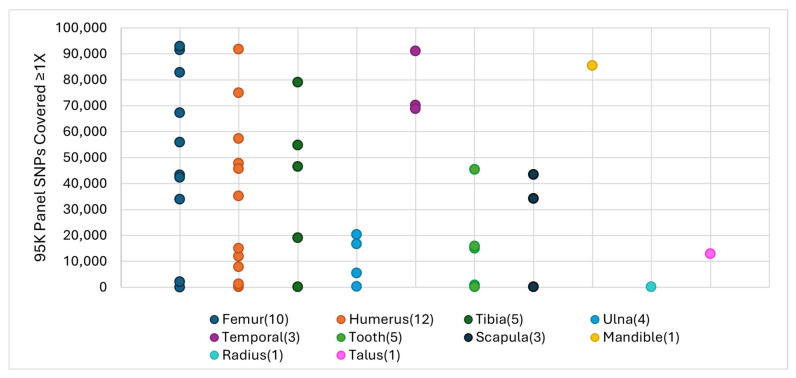
95K panel SNP recovery ≥1X per skeletal element. Numbers in parentheses next to each element represent the number tested. Each point represents one skeletal sample (*n* = 45).

A variety of skeletal elements were represented in the validation data set, although the skeletal element was unknown for some bone fragments (which were excluded from the skeletal element comparison). Due to historical preference for sampling long bones of the leg and arm [5,27,28,29], the most common elements tested were femurs (*n* = 10) and humeri (*n* = 12), which together represented 49% of all bones in the study. The femur and humerus samples generated moderate SNP recoveries on average (~48,000 and ~30,000 SNPs, respectively). While only three temporal bones were processed, their SNP recovery rate was consistently high relative to other elements, with an average of ~70,000 SNPs (Figure 4). A single mandible produced similarly high SNP recovery with 85,395 SNPs. By contrast, the five lower arm bones (one radius and four ulnas) resulted in comparatively low SNP recovery overall (<21,000 SNPs). As more 95K SNP data are generated in casework, SNP recovery will be tracked by skeletal element to determine the preferred bones for future sampling efforts at the DPAA.

#### 3.1.3. FRS Contamination Study

A total of 11 RBs and 36 NCs were included in the contamination assessment for reference sample processing. The 1X SNP recovery ranged from 10 to 90,343 SNPs with an average of 4947 SNPs. There were two contaminated control blanks, both of which were RBs, recovering 24,130 and 90,343 SNPs. These could be traced to the PC and a co-processed sample, respectively. To source contamination, kinship inference in Parabon Fx was used to compare the control blanks to other samples in the set. The most likely relationship was self for each contaminated RB when compared to a co-processed library, which suggested that the contamination was from a single source. The contamination observed in the RBs from simultaneously processed samples underscores the need for careful execution of the DNA extraction and manual library preparation procedure. Aside from contamination introduced during sample preparation (e.g., extraction, library preparation), it was suspected that cross-talk contributed to the SNP counts in the RB and NC data. In addition to higher 1X SNP recoveries than expected, relationships more distant than self were inferred between the control blanks and co-processed samples/PCs, suggesting more than one contributor. The majority of the FRS library sets were processed using single-indexed adapters, which are more susceptible to cross-talk than UDI adapters. In order to directly assess the impact of cross-talk, nine control blanks ranging from 2958 to 13,014 SNPs were resequenced without the associated samples. Resequencing reduced the SNPs recovered to a maximum of 321. Due to the cross-talk observed with the single-index adapters, subsequent testing was performed with UDI adapters and resulted in 1X SNP recovery <110 in control blanks (*n* = 8). Control blanks not heavily impacted by cross-talk, i.e., data generated from the resequencing event or with UDI adapters (*n* = 17), were used to calculate an analytical threshold using the average baseline signal plus ten standard deviations. The resulting analytical threshold was 1000 SNPs at ≥1X (Table S4). This analytical threshold (for FRS processing) distinguished clean control blanks and failed reference-type samples as those below 1000 SNPs (≥1X), while ≥1000 SNPs (≥1X) constituted an authentic signal for identifying contaminated control blanks and reference samples suitable for analysis.

#### 3.1.4. Skeletal Sample Contamination Study

Eighteen NCs and 17 RBs processed alongside skeletal sample sets, some sequenced at more than one multiplexing level, produced very few SNPs, with 94% generating <100 SNPs. The maximum 1X SNP recovery for an NC was 35 SNPs. There were two RBs with detectible human contamination that could have been attributable to co-processed samples, which produced 559 and 2981 SNPs. Excluding these two contaminated RBs, an analytical threshold for skeletal sample processing was calculated using the average number of SNPs covered ≥1X in RBs and NCs plus ten standard deviations. This resulted in an analytical threshold of 300 SNPs (Table S5). While a control blank with <300 SNPs (≥1X) would be considered clean, a skeletal sample that results in ≤300 SNPs would be considered failed.

### 3.2. Case-Type Sample Study

Based on the expectations set in the reference sample performance evaluation, 47 FRS sequence data files were included in the case-type sample study. The included FRS resulted in >75,802 (80%) SNPs at a 5X threshold, which corresponded to a range of 87,329–93,031 SNPs recovered at the 1X threshold. Any skeletal samples resulting in <300 SNPs were considered failed and were not included in the case-type sample study. Based on these criteria, 41 skeletal sample sequence data files were included in this study. Pairwise kinship analyses were performed between the 41 skeletal sample sequence data files and 47 FRS sequence data files for a total of 1927 comparisons, including both known relatives and unrelated individuals (Table S6). A relationship category between parent–child and 4th degree was expected in 62 of these pairwise comparisons (Table 2), leaving 1865 instances where unrelated was the expected most likely relationship category. Eight of the 1865 pairwise comparisons were between an SM and FRS more distantly related than 4th degree (four 6th degree and four 8th degree). Therefore, in this context, the expected relationship was unrelated for these comparisons.

Based on inferred global ancestry proportions in Parabon Fx, 75.6% (31) of the skeletal sample sequence data files were from individuals of European ancestry (i.e., >90% inferred European ancestry). One sample was from an SM of primarily Native American ancestry. The remaining nine samples were from SMs that were of admixed ancestry: three were admixed between African and European ancestry (African American), and six were a combination of European, Native American, and/or African/Asian ancestry (Latino). This ancestry distribution is consistent with US SMs from past conflicts, typical of the AFMES-AFDIL casework. No skeletal samples from individuals of non-admixed African, East Asian, or Central/South Asian ancestry were included in the case-type sample study due to a lack of availability.

#### 3.2.1. Related Pairs

For related pairs, defined here as 4th degree relatives and closer, 60 of 62 kinship analyses resulted in a most likely relationship category that was consistent with the expected relationship. There were two instances in which the inferred relationship was unexpected. In one of the instances, the most likely relationship category was 4th degree while the expected was 3rd degree. In this case, only 383 SNPs were recovered for the skeletal sample, and low SNP recovery is likely causing the unexpected result. The other instance initially appeared to be a false negative, where Parabon Fx inferred that the two individuals were unrelated when a 2nd degree relationship was expected. This FRS had not been previously tested for casework, as it was not suitable for mitogenome testing. Additionally, Y-STR testing was not completed for the FRS due to the degraded nature of the DNA in the associated skeletal sample. Given the relatively high SNP recovery of the skeletal sample (78,121 SNPs), the genealogy of the SM and FRS was reassessed. Unfortunately, no conclusive genealogical documentation was available for the FRS, presumed to be the grandson of the SM. However, two other FRS related to the same SM were tested, and the expected relationships were consistent with those inferred, confirming the identity of the SM. It is therefore suspected that a misattributed paternity event occurred in the pedigree, resulting in the SM being biologically unrelated to this particular FRS. For the remaining analyses in the case-type sample study, the unexpected inference involving the low SNP recovery sample was included, but the unexpected inference due to the suspected pedigree issue was excluded.

Overall, the average log10 LR of the most likely relationship category decreased as the genetic distance increased (Table 3). While the skeletal sample SNP recovery range varied between relationship categories, when the most likely relationship was 3rd degree or closer, the log10 LR was above the threshold of 4. This included a 3rd degree inference involving a skeletal sample with only 2400 SNPs. However, not all comparisons with 4th degree as the most likely relationship category resulted in a log10 LR above the threshold. There was one instance of a skeletal sample with fewer than 2000 SNPs (1989 SNPs) where the inferred relationship category was consistent with the expected. However, this was a 4th degree relationship with a log10 LR of 0.67, which would be considered inconclusive.

Aside from genetic distance, the number of SNPs recovered, and, by extension, the number of overlapping SNPs after LD pruning, clearly affected the log10 LRs within a given relationship category (Table 3). The overlapping SNPs (i.e., those recovered in both the skeletal sample and FRS being compared) after LD pruning are used for the calculation of kinship analysis statistics, including the LRs. Of note, high SNP recovery (>75,802 SNPs ≥5X) was required for the FRS to be included in the case-type sample study. Therefore, the skeletal sample SNP recovery was indicative of the number of overlapping SNPs after LD pruning (Table 3 and Figure S1). While the overlapping SNPs after LD pruning are directly tied to kinship analysis statistics, the results below are presented with respect to skeletal sample SNP recovery. This analysis is informative for the assessment of when strong statistical support may be expected for kinship inferences in skeletal sample processing. Given the relationship between skeletal sample SNP recovery and overlapping SNPs after LD pruning (Figure S1), trends observed in the data would be analogous.

**Figure 5:**
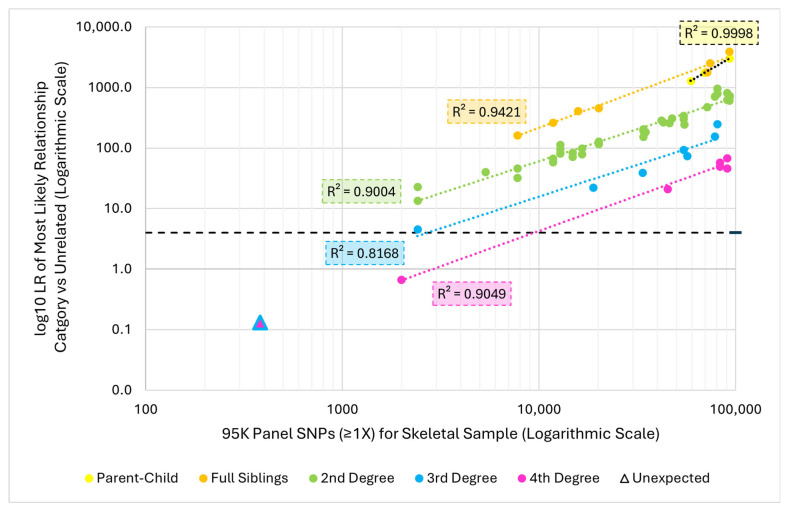
Relationship between 95K panel SNPs recovered ≥1X from the skeletal sample and the log10 likelihood ratio (LR) of the most likely relationship category (versus unrelated) for pairwise kinship analyses between related pairs (*n* = 61). The unexpected relationship inference is represented by a triangle where the main color is the inferred relationship category, and the outline is the expected relationship category. The black dashed line represents the minimum log10 LR threshold of 4. Trendlines and associated R^2^ values for each relationship category are present in the corresponding color indicated in the legend, aside from the trendline for Parent–Child, which is black.

In order to further investigate the impact of skeletal sample SNP recovery on the resulting LRs, the log10 LR of the most likely inferred relationship category for each comparison was plotted against the number of SNPs recovered ≥1X for the skeletal sample (Figure 5). Within each relationship category, increased SNP recovery was positively correlated with higher log10 LRs (Figure 5). Also, to reach a given LR, fewer SNPs were needed for closer relationships than for more distant ones. For example, ~20,000 SNPs resulted in a full sibling inference with a log10 LR of 456, but only a log10 LR of 116 for a 2nd degree inference. In fact, over 71,000 SNPs were needed to infer a 2nd degree relationship with a similar log10 LR (475). Considering comparisons with high recovery skeletal samples (>80,000 SNPs), the maximum log10 LR observed for 1st degree relationships was over 3000, while 2nd degree relationships reached 960, 3rd degree up to 248, and 4th degree only 68 (Table 3). Regardless of the relationship category, with at least 2400 SNPs, the most likely relationship category was consistent with the expected relationship and exceeded the log10 LR threshold of 4. No false negatives were observed as no related pairs resulted in unrelated as the most likely relationship category.

**Figure 6:**
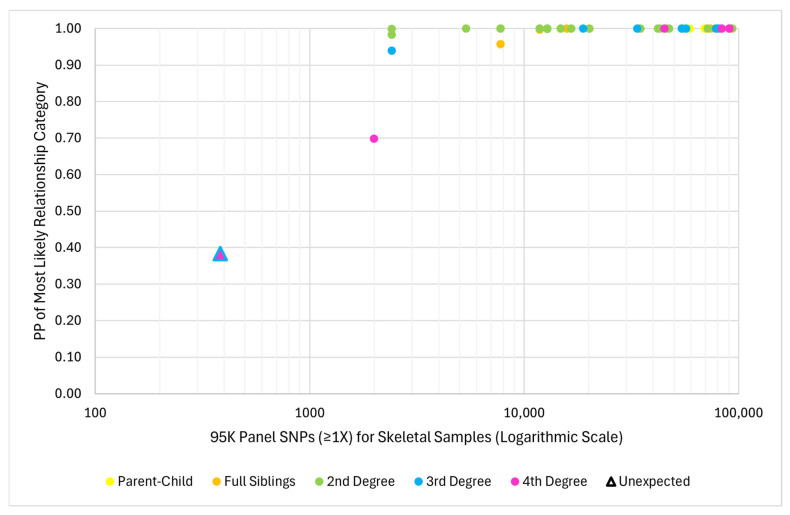
Relationship between 95K panel SNPs recovered ≥1X from the skeletal sample and the posterior probability (PP) of the most likely relationship category for pairwise kinship analyses between related pairs (*n* = 61). The unexpected relationship inference is represented by a triangle where the main color is the inferred relationship category, and the outline is the expected relationship category.

Two PP thresholds, 95% and 99.99%, were also applied for the interpretation of the most likely inferred relationship category to lend statistical weight to the LR of that category relative to the others. The unexpected inference (which included the skeletal sample with 383 SNPs and yielded a most likely inference one degree more distant than the expected relationship) resulted in a PP of 0.38% for the most likely relationship category. This instance was then considered inconclusive (with a PP <95%). Two other inferences were consistent with the expected relationship category but resulted in PPs <95%, also rendering them inconclusive (one 3rd degree with a PP of 93.99% and one 4th degree with a PP of 69.92%). Both inferences included skeletal samples with <2500 SNPs recovered. All remaining inferences resulted in a PP <95% for the most likely category. The minimum number of skeletal sample SNPs that resulted in a PP ≥95% was 2400 (a 2nd degree inference), and the minimum for a PP ≥99.99% was 5300 (also a 2nd degree inference). Regardless of relationship category, when 1X SNP recovery of the skeletal sample was >10,000, the PP was ≥99.99% in all but one instance (99.982%, 2nd degree inference for a skeletal sample with 12,820 SNPs) (Figure 6). Therefore, when the skeletal sample SNP recovery was >13,000 SNPs, the PP was consistently ≥99.99%.

After applying the log10 LR threshold of ≥4 and a PP threshold of ≥99.99%, a total of eight related pairs resulted in an inconclusive inference (Table 4). Therefore, after applying these thresholds for strong statistical support, all kinship inferences that exceeded both PP thresholds were consistent with expected relationships that could be confirmed with genealogy.

Two FRS, which were 6th and 8th degree relatives of the same SM, were intentionally included in the validation despite being beyond the degree of relatedness that Parabon Fx can currently infer with capture-to-capture kinship analysis. These two FRS were compared to four different skeletal samples from the associated SM, generating eight pairwise comparisons. All resulted in most likely inferences of unrelated with PP <99.99%, which was the expected outcome.

#### 3.2.2. Unrelated Pairs

For the remaining 1865 pairwise comparisons between unrelated pairs (skeletal samples versus unrelated FRS), the most likely relationship was unrelated for 85.1% of the inferences (1588 comparisons) (Table 5). With a PP threshold of ≥95%, 79.1% (1476) of the unrelated inferences reached strong statistical support, while 65.8% (1227) of the inferences reached the 99.99% threshold. The seemingly high number of inconclusive inferences for unrelated pairs may be due in part to the consideration of all tested relationships when calculating the PP, rather than only the expected relationship and unrelated. It was observed that higher skeletal sample SNP recovery (>60,000 SNPs) was needed for an unrelated inference to consistently meet these PP thresholds (Figure S2) compared to the other relationship categories (Figure 6). Aside from the unrelated inferences, the other 14.9% (*n* = 277) of the comparisons were either inconclusive or false positives (Table 5). One 2nd degree inference exceeded the log10 LR threshold of 4 (11.8). This single 2nd degree inference involved an admixed (Latino) skeletal sample with only 2412 SNPs compared to a European FRS, and the PP for the 2nd degree relationship was 99.79%. Therefore, with a PP threshold of 95%, this inference would be considered a false positive, but a threshold of 99.99% would render it inconclusive.

The most likely relationship category was 4th degree in 91.7% of inferences that resulted in a most likely relationship category other than unrelated (254 of 277) (Table 5). One third (84) of the 4th degree inferences had a PP <95% and were considered inconclusive. There were 170 instances (66.9%) where the PP was ≥99.99% (all instances with log10 LRs <4), which were considered to have strong statistical support and deemed false positives. These false positive 4th degree inferences originated from six skeletal samples representing four SMs, all with admixed ancestry. Two samples from an African American SM were involved in the majority (76%) of the false positive inferences (Table 6; samples 1578-1 and 1578-2), while 23% were from one sample from a SM of Latino ancestry (sample 1581-1). Two skeletal samples from another Latino SM produced only one false positive each. Both skeletal samples resulted in 1X SNP recovery <35,000 (Table 6; 1563-3 and 1563-4). Only one false positive was observed between a skeletal sample and FRS that, while unrelated, were both of the same ancestry (Latino). In this case, the Parabon Fx ancestry inferences for the skeletal sample and FRS resulted in 75 to 85% European ancestry, but both were considered admixed using the <90% single global ancestry threshold applied here. All remaining false positives with strong statistical support were between a skeletal sample from an admixed SM and an FRS of a differing ancestry, with at least one global ancestry population shared between them, namely European (Table 6). In 94% of instances, the FRS was European (>90% European) (Table S7). The remaining ten instances involved skeletal samples from SMs of African American ancestry being compared to FRS of a different admixed ancestry, either Latino or Asian American, both of which include a portion of European ancestry. In order to determine if the allele frequency file for the majority ancestry of the skeletal sample would be more appropriate than the admixed file, kinship analyses were rerun. However, this produced more false positives with strong statistical support (log10 LR ≥4 and PP ≥99.99%) or inferred relationships closer than 4th degree with strong statistical support (Table S8). These results indicate that the admixed allele frequency files are more representative of the admixed individuals tested than the ancestry file for the highest proportional global ancestry. Of note, the other four admixed samples included in this study did not result in any false positives (Tables S8 and S9).

These results highlight the challenges of kinship analyses that incorporate population-specific allele frequencies when comparing samples of differing ancestries, as the selected allele frequency file may not be the most appropriate for one of the two individuals. It is possible that improving the representation, both in number and proportional global ancestries, of admixed individuals in datasets used to create the kinship allele frequency files may reduce false positive results to a degree. While the allele frequency files for African, Central/South Asian, East Asian, and European groups were created with >480 individuals each, the admixed African American and Latino allele frequency files only included 141 and 315 individuals, respectively. However, it is difficult to create a representative admixed file, particularly for individuals in the U.S., when the ancestry proportions will differ from person to person and by geographic location. It is worth noting that the allele frequency file used has a limited impact on direct comparisons (i.e., kinship inference of self) and comparisons between 1st degree relatives, which is a common scenario in most forensic laboratories.

### 3.3. Efficiency-Gain Modifications

Due to the cost and time requirements for the lab protocols described herein, potential efficiency gains were assessed both during and after the completion of the main validation studies.

#### 3.3.1. Combined/Pooled Captures

The 95K panel is a custom SNP panel with 180,000 unique baits, which makes it more expensive than pre-designed myBaits panels. For time and cost savings, Arbor Biosciences suggests in their myBaits user manual to pool sample libraries prior to hybridization capture when the samples are of relatively high quality, such as FRS. Pooled capture reduces sample library consumption, as well as cost and processing effort per sample. Full sets of FRS, including associated controls, were combined in equal volume prior to capture, with a minimum of four libraries and a maximum of 13 libraries per pool (including associated controls). Each FRS set’s library pool was then enriched in a single myBaits 95K capture reaction.

When at least 70% of 95K SNPs were recovered ≥5X in individual captures of the FRS, which is expected for high-quality reference samples, SNP recovery from pooled FRS captures was found to be comparable (Table 7). There were three instances where SNP recovery in the pooled capture was substantially reduced compared to the individual captures, two of which were samples with >400 ng total DNA input into library preparation. The overloaded library preparation reactions likely resulted in poorer library conversion success, ultimately reducing SNP recovery for those FRS when competing with other samples during capture. Limiting the DNA input into library preparation to a standard quantity could positively impact sample success by effectively normalizing the FRS, which would be particularly helpful when DNA extracts of varying concentrations are processed together.

#### 3.3.2. Off-Target Mitogenome Analysis

Mitogenome analysis has historically been a primary testing modality for the Past Accounting Section, as mtDNA is typically of sufficient quantity and quality for analysis when STR testing fails, is a quality control check of the casework DNA sample, allows for efficient sorting of commingled remains, and allows for probative searching of the existing AFMES-AFDIL FRS mtDNA database. Therefore, FRS that are appropriate mitogenome references have mtDNA control region data (from Sanger sequencing performed prior to the validation of an NGS method in 2016) or undergo mitogenome sequencing via NGS. For those FRS without mtDNA data in the AFMES-AFDIL FRS database (or only control region data from pre-NGS processing), gaining full mitogenome sequence information concurrently with SNP data would prevent additional lab processing. In order to determine the amount of off-target mitogenome data that can be gained from 95K-captured FRS libraries, the same paired FASTQ files analyzed in Parabon Fx were also analyzed using the AFMES-AFDIL validated mitogenome analysis workflow utilized for analysis of mitogenome capture data. The subset of FRS tested for this efficiency gain experiment varied in SNP recovery, yet all produced complete mitogenome profiles with average coverage of the mitogenome ranging from 800X to 17,000X (Table S10). These results indicate that it is not necessary to do separate mitogenome processing of 95K-captured FRS in order to generate valuable mtDNA sequence data.

The off-target mitogenome reads were also analyzed for a set of skeletal samples. As expected, the proportion of the mitogenome covered ≥5X varied with skeletal sample quality. The number of 95K SNPs covered at ≥5X was a useful indicator of off-target mitogenome success. If ≥70% of the 95K SNPs (~66,000) were covered at ≥5X, off-target mitogenome average coverage was over 60X. At the other extreme, if <0.1% of the 95K SNPs (~95) were covered at ≥5X, off-target mitogenome average coverage was <5X and thus below the validated threshold for reporting mtDNA profiles (Table S11). While this approach may be sufficient for some skeletal samples, additional modifications seek to improve the mtDNA recovery from SNP-captured case sample libraries.

#### 3.3.3. SNP Capture with Mitogenome Bait Spike-In

Skeletal samples with low SNP recoveries did not result in sufficient off-target mtDNA data for library single source assessment and/or haplotype comparisons. Therefore, a future modification to the hybridization capture enrichment of skeletal samples, with baits for both 95K SNPs and the mitogenome included in the same reaction, would allow for simultaneous targeting of both rather than requiring two separate captures. This would substantially reduce processing cost and time. An added benefit of a SNP/mitogenome spike-in capture would be that it also conserves library product, leaving more for testing with other bait panels, depending on the need in each particular scenario.

## 4. Conclusions

This validation showed that hybridization capture enrichment of both skeletal samples and associated FRS with the 95K SNP panel can result in successful kinship inference for a range of ancestries and expected relationships. FRS with at least 7 ng of DNA input into library preparation resulted in an average of 82.5% of the 95K SNPs with at least 5X coverage and 95.2% at ≥1X coverage. SNP recovery from skeletal sample extracts was difficult to predict due to the degree of degradation, damage, and environmental/microbial DNA content, which varied widely between samples. No false negative inferences were observed as no related pair (out to 4th degree) resulted in unrelated as the most likely relationship category regardless of SNP recovery. If >13,000 SNPs were recovered at ≥1X coverage in a skeletal sample, kinship analysis with associated FRS resulted in a most likely relationship category consistent with the expected relationship backed by strong statistical support (log10 LR ≥4 and PP ≥99.99%). In the future, log10 LR thresholds may be tailored to each relationship category as they varied by degree of relatedness and skeletal sample SNP recovery. Pairwise kinship analysis between unrelated individuals yielded an unrelated inference that met the ≥99.99% PP threshold when the skeletal sample resulted in at least 60,000 SNPs ≥1X. There were instances of false positive 4th degree relationships with strong statistical support. All but one of the false positives occurred in cases where a skeletal sample from an individual of admixed ancestry was compared to an FRS of a different ancestry, although not all admixed individuals produced false positives. As a result of this finding, additional lines of DNA evidence will be required to support a 4th degree kinship inference when admixture is observed, such as lineage marker analysis (mtDNA or Y-chromosomal DNA).

The validation of the 95K SNP panel was completed in December 2024, and implementation into casework occurred in early 2025. The first DPAA identification assisted by SNP testing was announced in March of 2025 [30]. As of 1 October 2025, over 213 skeletal samples and 531 FRS have been processed with 95K SNP capture, leading to 86 new identifications, enabling DPAA to make more than 200 identifications for fiscal year 2025. SNP processing is expected to become a leading tool for DPAA casework at the AFMES-AFDIL.

Each of these identifications has a story, such as that of Ensign Mandeberg [30,31]. On 15 August 1945, a formation of six US aircraft in Navy Fighter Squadron 88 was launched for a fighter sweep near Tokyo, Japan. Before arriving, the formation received a radio transmission stating the Empire of Japan had surrendered, effectively ending the war. As they were returning to the USS *Yorktown* on which they were stationed, they were attacked by approximately 20 Japanese fighter planes near Atsugi Airfield. Four of the six Hellcats never returned, and the four pilots aboard those aircraft were reported Missing in Action that day. During the following year, remains were recovered near the wreckage of a US aircraft with partial markings linking it to the USS *Yorktown* and identifying it as the plane flown by Ensign Eugene Mandeberg. While excellent wartime research led to circumstantial evidence that strongly suggested the remains were those of Mandeberg, the fragmentary state of the remains made a positive medical identification impossible. Navy officials honored the request of Mandeberg’s family at that time to inter the remains as a World War II Unknown at the Manila American Cemetery and Memorial [32].

In 2019, these remains were exhumed for DNA testing and comparison with two FRS that had been collected for Mandeberg. At that time, NGS testing of the mitogenome produced results consistent with the mtDNA FRS for Mandeberg and excluded two of the possible SMs. However, the fourth SM could not be excluded because he did not have an mtDNA reference available for comparison. Autosomal and Y-STR testing of the skeletal samples was attempted but failed to generate usable data due to the degraded nature of the DNA. Although DPAA and the military continued to search for a family member who could be a mtDNA reference for the fourth SM, this case remained stalled during the development and full validation of a 95K SNP method that was amenable to such challenging samples and available FRS. When SNPs were implemented in 2025, two different skeletal elements from this case were among the first tested. Extended kinship inferences from the SNP data allowed for the exclusion of the fourth SM and generated strong statistical support for the expected relationships with family members of Ensign Mandeberg. His identification occurred on 4 March 2025, and Ensign Mandeberg became the first U.S. military identification made using SNPs, supported by anthropological and historical research. Autosomal SNP testing made Ensign Mandeberg’s identification possible, along with many others like it, creating a new era in DNA-assisted identifications of America’s missing military heroes.

## Figures and Tables

**Table 1:** Multiplexing ranges used for NextSeq 550 sequencing based on sample type and sequencing kit. The total number of libraries includes associated controls. Theoretical reads per library indicate the expected number of reads given a uniform distribution between libraries corresponding to the multiplexing range. M—million.

Sample Type	Library Preparation Kit	Sequencing Kit (Cycling Approach)	Read Output	Multiplexing Range–Total Number of Libraries (Theoretical Reads per Library)
FRS	HyperPlus	Mid Output 300-cycle v2.5 (151 + 151)	260 M	14–27 (18.6 M–9.6 M)
Skeletal	HyperPrep	Mid Output 150-cycle v2.5 (76 + 76)	260 M	7–26 (37.1 M–10.0 M)
		High Output 150-cycle v2.5 (76 + 76)	800 M	26–54 (30.8 M–14.8 M)

**Table 2:** Relationships represented for related pairs in the case-type sample study. The double line border separates relationship categories that Parabon Fx can infer (parent–child to 4th degree) (*n* = 62) from those more distant (*n* = 8).

Relationship Category	Number of Relationship Pairs
Parent–Child	3
Full Siblings	7
2nd Degree	38
3rd Degree	8
4th Degree	6
6th Degree	4
8th Degree	4

**Table 3:** Minimum (Min), maximum (Max), and average (Avg) log10 likelihood ratio (LR) of the most likely relationship category versus unrelated for all pairwise kinship inferences between related pairs. Inferences consistent with the expected relationship are included (*n* = 60) while unexpected results are excluded (*n* = 2). LD—Linkage disequilibrium.

Relationship Category	Count	Value	Skeletal Sample 95K Panel SNP Recovery (≥1X)	Overlapping SNPs After LD Pruning (≥1X)	Log10 LR vs. Unrelated
Parent–Child	3	Min	59,059	44,872	1282.52
		Max	93,001	61,582	3021.79
		Avg	73,817	52,400	2021.83
Full Sibling	7	Min	7755	7379	161.81
		Max	92,984	63,195	3916.67
		Avg	41,987	31,359	1363.52
2nd Degree	37	Min	2412	2362	13.45
		Max	93,001	56,918	961.85
		Avg	41,394	31,128	295.87
3rd Degree	7	Min	2412	2368	4.52
		Max	80,486	57,091	248.92
		Avg	46,348	35,091	91.11
4th Degree	6	Min	1989	1935	0.67
		Max	90,433	60,145	68.49
		Avg	65,700	46,014	40.66

**Table 4:** Number of inferences for each relationship category exceeding the 95% and the 99.99% posterior probability (PP) thresholds for related pairs. All inferences included met the log10 likelihood threshold of ≥4. The * indicates that one of the two inconclusive results is an instance where the most likely relationship was inconsistent with the expected relationship.

Most Likely Relationship Category	Expected Number	Number with PP ≥95%	Number with PP ≥99.99%	Number Inconclusive (PP <99.99%)
Self	0	0	0	0
Parent–Child	3	3	3	0
Full Sibling	7	7	5	2
2nd Degree	37	37	34	3
3rd Degree	8	6	6	1
4th Degree	6	5	5	2 *
Unrelated	0	0	0	0
Total (Percent)	61	58 (95%)	53 (87%)	8 (13%)

**Table 5:** Kinship inference results from 1865 pairwise comparisons between samples expected to be unrelated. All comparisons were between a skeletal sample and a reference sample. A log10 likelihood ratio threshold of ≥4 was applied.

Most Likely Relationship Category or Inconclusive	Total Inferences		Inferences with PP ≥95%		Inferences with PP ≥99.99%	
	Number	Percentage	Number	Percentage	Number	Percentage
Self	0	0%	0	0%	0	0%
Parent–Child	0	0%	0	0%	0	0%
Full Sibling	0	0%	0	0%	0	0%
2nd Degree	4	0.2%	1	0.1%	0	0%
3rd Degree	19	1.0%	0	0%	0	0%
4th Degree	254	13.7%	170	9.1%	170	9.1%
Unrelated	1588	85.1%	1476	79.1%	1227	65.8%
Inconclusive	N/A	N/A	218	11.7%	468	25.1%

**Table 6:** Skeletal samples yielding false positive kinship results with strong statistical support (log10 likelihood ratio ≥4 and PP ≥99.99%). All were 4th degree inferences. The first four digits of the Skeletal Sample name indicate the service member, and the number after the dash indicates the skeletal sample tested. The kinship allele frequency file used was based on skeletal sample inferred ancestry. Differing Ancestry refers to the reference sample ancestry that is different from that of the skeletal sample. All reference samples involved in these false positives included at least 12% European ancestry. AfAm-African American.

Skeletal Sample	1X 95K Panel SNPs	# False Positives	Skeletal Sample Ancestry					Kinship Allele Frequency File Used	Proportion of References	
			Africa	America (Native American)	Central/South Asia	East Asia	Europe		With Differing Ancestry	With <75% European Ancestry
1563-3	33,608	1	10.86%	32.82%	0.00%	0.00%	56.32%	Latino	100%	100%
1563-4	18,848	1	10.49%	31.61%	0.00%	0.00%	57.91%	Latino	100%	100%
1575-1	71,488	44	71.03%	0.00%	0.00%	0.00%	28.97%	AfAm	100%	95.5%
1578-1	47,322	43	77.70%	0.00%	0.00%	0.00%	22.30%	AfAm	100%	97.7%
1578-2	42,884	42	78.72%	0.00%	0.00%	0.00%	21.28%	AfAm	100%	97.6%
1581-1	80,486	39	0.00%	10.27%	4.73%	0.00%	85.00%	Latino	97.4%	100%

**Table 7:** Matrix of the percentage (and number) of samples associated with each scenario in the comparison of individual sample 95K panel SNP capture reactions to pooled/combined 95K captures. The 95K panel includes 94,752 total SNPs; thus, 70% corresponds to 66,326 SNPs.

		Individual Capture 95K Panel SNP Recovery (≥5X Coverage)	
		≥70% of SNPs	<70% of SNPs
**Pooled Capture 95K Panel SNP** **Recovery (≥5X Coverage)**	**≥70% of SNPs**	89.0% (*n* = 65)	0% (*n* = 0)
	**<70% of SNPs**	4.1% (*n* = 3)	6.9% (*n* = 5)

## Data Availability

The data presented in this study are stored at the AFMES-AFDIL and may be made available to approved laboratories upon written request to the corresponding authors due to privacy restrictions.

## Supplementary Materials

The following supporting information can be downloaded at https://www.mdpi.com/article/10.3390/genes17030306/s1. Table S1: Skeletal sample details; Table S2: Family reference sample details; Table S3: Details about reference datasets for ancestry and kinship allele frequency files in Parabon Fx; Table S4: Threshold calculation for FRS processing using negative controls (NC) and reagent blanks (RB); Table S5: Threshold calculation for skeletal sample processing using negative controls (NC) and reagent blanks (RB); Table S6: Number of kinship inferences run with each allele frequency file (FF) in Parabon Fx for the 95K panel; Figure S1: Number of 95K SNPs recovered ≥1X for the skeletal sample (x-axis) compared to the number of 95K SNPs recovered ≥1X shared between the skeletal sample and FRS being compared (overlapping SNPs) after linkage disequilibrium pruning in kinship analysis between related pairs; Figure S2: Number of 95K SNPs recovered ≥1X for the skeletal sample (x-axis) compared to the posterior probability for most likely relationship inferences of unrelated; Table S7: False positive comparisons with strong statistical support (log10 LR ≥4 and PP ≥0.9999); Table S8: False positive comparisons with strong statistical support (log10 LR ≥4 and PP ≥0.9999) with admixed allele frequency file and majority ancestry frequency file; Table S9: Admixed Skeletal Samples versus unrelated FRS resulting in a most likely relationship of unrelated with PP <95%; Table S10: Average coverage of the mitogenome (mtG) from off-target mtG analysis of FRS captured with 95K panel baits; Table S11: Average coverage of the mitogenome (mtG) from off-target mtG analysis of skeletal samples captured with 95K panel baits.

## Abbreviations

The following abbreviations are used in this manuscript:

25K	25,000 SNP panel
95K	95,000 SNP panel
AfAm	African American
AFMES-AFDIL	Armed Forces Medical Examiner System’s Armed Forces DNA Identification Laboratory
AQME	AFDIL-QIAGEN mtDNA Expert plug-in
DPAA	Defense POW/MIA Accounting Agency
DNA	Deoxyribonucleic acid
EDTA	Ethylenediaminetetraacetic acid
FIGG	Forensic Investigative Genetic Genealogy
FRS	Family reference sample
GL	Genotype likelihood
GRCh38	Genome Reference Consortium human reference genome assembly 38
IBD	Identical by descent
LD	Linkage disequilibrium
LR	Likelihood ratio
mtDNA	Mitochondrial DNA
mtG	Mitochondrial genome/Mitogenome
NC	Negative control
NGS	Next-generation sequencing
PC	Positive control
PCR	Polymerase chain reaction
PP	Posterior probability
RB	Reagent blank
rCRS	Revised Cambridge Reference Sequence
SM	Service member
SNP	Single nucleotide polymorphism
STR	Short tandem repeat
UDI	Unique dual-indexed
US	United States
USS	United States Ship
WWII	World War II
